# Influence of blood glucose fluctuation, C-peptide level and conventional risk factors on carotid artery intima–media thickness in Chinese Han patients with type 2 diabetes mellitus

**DOI:** 10.1186/s40001-019-0370-0

**Published:** 2019-02-20

**Authors:** Min Liu, Li Ao, Xinyu Hu, Jianning Ma, Kena Bao, Ye Gu, Jing Zhao, Weiping Huang

**Affiliations:** 1 Department of Scientific Research, Jiading District Central Hospital Affiliated Shanghai University of Medicine & Health Sciences, Shanghai, China; 2 Department of Nursing, Jiading District Central Hospital Affiliated Shanghai University of Medicine & Health Sciences, Cheng Bei Road, Jiading District, Shanghai, 201800 China; 3 The Former Dalian Sanatorium of Shenyang Military Region, Dalian, Liaoning China

**Keywords:** Blood glucose fluctuation, CIMT, T2DM

## Abstract

**Background:**

Some studies have suggested that blood glucose fluctuation and C-peptide level were considered as predictive factors for carotid artery intima–media thickness (CIMT). However, the relationships of these variables are unclear. This research was aimed to identify the potential effects of blood glucose fluctuation, C-peptide level and conventional risk factors on CIMT.

**Methods:**

A total of 280 type 2 diabetes mellitus (T2DM) patients were enrolled into this study. Population characteristics were obtained through medical history and clinical parameters. The patients were divided into two groups according to the critical value of CIMT (0.9). Research data were analyzed to identify risk factors of CIMT between the two groups.

**Results:**

The comparison results of basic information showed that differences in age and illness years between the two groups were statistically significant (*p* = 0.0002 and *p* = 0.0063). Logistic regression analysis results indicated that smoking, uric acid (UA) levels, 2 h C-peptide and standard deviation of blood glucose (SDBG) were the influence factors for CIMT thickening (*p* = 0.032, *p* = 0.047, *p* = 0.049 and *p* = 0.042, respectively). Blood glucose fluctuation could affect the risk of some complications. In largest amplitude of glycemic excursions (LAGE) > 4.4 group, the CIMT abnormal rate was 27.10%, which was significantly higher than 12.12% in the LAGE ≤ 4.4 group (*p* = 0.012). The CIMT abnormal rate of SDBG > 2.0 group was 27.81%, which was significantly higher than that of the SDBG ≤ 2.0 group (*p* = 0.018).

**Conclusions:**

Blood glucose fluctuation is an independent risk factor associated with CIMT in T2DM patients, in addition to conventional risk factors, such as smoking, high UA level and 2 h C-peptide. Therefore, more attention should be given to the change of CIMT and the complications.

## Introduction

Diabetes mellitus (DM) is a serious chronic and harmful disease, which threatens human health all over the world [1]. The incidence of type 2 diabetes mellitus (T2DM) increases rapidly because of unbalanced diet, changes in life style and aging [2]. According to the survey results of the International Diabetes Federation (IDF), there were about 382 million diabetes patients worldwide in 2013 [3]. The World Health Organization’s 2016 overview of diabetes in China pointed out that diabetes accounts for 2% of the proportion of death causes in all age groups. It seems that the mortality rate of diabetes is not very high, but human health is threatened by its complications seriously, such as cardiovascular diseases and cancers, which account for 45% and 23% of all death causes, respectively. Diabetes and its complications have a great economic impact on individuals, families, health systems and countries [4, 5].

At present, the pathogenesis of diabetic chronic complications has not been thoroughly clarified. It may be affected by genetic susceptibility, insulin resistance, hyperglycemia, inflammatory reaction and other factors [6–8]. Cardiovascular disease and cerebrovascular disease are the major complications of diabetes [9]. It is reported that the incidence of cardiovascular diseases in diabetes patients is two to four times higher than that in non-diabetes patients. Vascular pathologies account for 60–80% of all death causes in diabetes patients [10–12]. Carotid intima–media thickness (CIMT) can reflect the degree of early artery atherosclerosis, which is a crucial method to detect early arterial intima damage [13–16]. Studies have shown that abnormal fasting and postprandial blood glucose are associated with CIMT [17, 18]. Recent studies suggest that abnormal fluctuation of blood glucose can also affect CIMT and other complications significantly [19–21]. Standard deviation of blood glucose (SDBG), the largest amplitude of glycemic excursions (LAGE) and postprandial glucose excursions of three dinners (PPGE) are common indicators of blood glucose fluctuation indicators. In this study, simple finger glucose fluctuation indexes were used to evaluate the glycemic fluctuation, study the influence of finger glucose fluctuation and traditional factors on CIMT.

Some studies indicate that C-peptide causes human atherosclerotic lesions in diabetic patients, because it induces smooth muscle cell proliferation [22, 23]. It is necessary to investigate the relationship among blood glucose fluctuation, C-peptide level and CIMT. It is also quite crucial to identify other risk factors of diabetic macroangiopathy from the basic information and medical history. Early diagnosis and treatment of vascular lesions in type 2 diabetes could improve the quality of life and prolong the survival times. Therefore, it is particularly important to identify the risk factors and sensitive predictors of diabetic macroangiopathy. This may provide new strategy for prevention and treatment of diabetes.

## Methods

### Study population and inclusion criteria

A total of 280 Chinese patients with type 2 diabetes, who were hospitalized in Shanghai Jiading District Central Hospital from January 2016 to December 2017, were recruited in this study (mean age = 59.85 [SD = 14.46], sex ratio man/woman = 181/99). The diagnosis of type 2 diabetes mellitus was based on the WHO diagnostic criteria in 2013. All the enrolled patients were of Han nationality and had no diabetic ketosis or nonketotic hyperosmolar coma. The patients in the following conditions were excluded: severe hepatic and renal dysfunction, heart deficiency and serious infection. The basic information such as age, gender, body mass index (BMI), smoking status, family history and other medical history were collected to identify risk factors of CIMT. All patients agreed to participate in this study and signed the medical informed consent. Meanwhile, this study was also approved by our local ethics committee.

### Research methods and biochemical examination

Venous blood samples were obtained through venipuncture. Then the blood samples were freezed (− 70 °C) to test the levels of fasting blood glucose (FBG), 2-h post-load glucose, C-peptide levels, creatinine, HbA1c, uric acid (UA), serum cholesterol, HDL-cholesterol, LDL-cholesterol, triglyceride (TG) and so on. Serum biochemicals were measured by automatic biochemical analyzer (Roche D/P/ISE, Switzerland) and HbA1c was measured by high-performance liquid chromatography (HLC-723g7, Japan).

Patients’ finger blood glucose levels before and after three meals and before sleep were recorded to calculate sugar fluctuation parameters including PPGE, LAGE and SDBG within 24 h after admission. PPGE is equal to the mean value of the absolute differences between the blood glucose level 2 h after each meal and the corresponding pre-prandial blood glucose level. LAGE corresponds to the differences between the maximum and minimum blood sugar levels of a day. SDBG is the standard deviation of the total blood glucose within 1 day.

### CIMT measurement

CIMT is located on the vertical dimension between carotid intima and media. It was measured by color Doppler flow imaging with a probe of 9–12 MHZ (CDFI, GE Vivid E9, Norway). Bilateral carotid arteries of patients were checked by a professional ultrasound doctor. The measurements were repeated three times and the average value was recorded. When CIMT ≥ 0.9 mm, it was considered to be abnormal according to the “the guidelines for prevention and control of hypertension”.

### Statistical method

Data consistent with the normal distribution and homogeneity of variance are presented as mean ± SD. The skew distributional data are expressed as median (IQR). Enumeration data and categorical variables were compared by Chi square analysis or Fisher’s exact test. Differences of continuous variables were tested by Student’s *t* test or analysis of variance (ANOVA). The possible relationships between CIMT and variables were evaluated by logistic linear regression analysis. The multivariable-adjusted model was used, including age, sex, BMI, smoking, the presence or absence of hypertension, cerebral infarction, HDL cholesterol, LDL cholesterol, triglycerides, creatinine, serum urea, uric acid, C-peptide level, homocysteine and HbA1c. It can give an assessment of the independent association of each possible variable and CIMT. *p* < 0.05 was considered as an indicator of significant difference. Data were evaluated by the software STATA version 12.0 (STATA Corp., College Station, Tex).

## Results

### Basic information

A total of 280 T2DM patients were enrolled in this research. The study population was divided into CIMT ≥ 0.9 group and CIMT < 0.9 group according to the “2013 European Guidelines for Hypertension Management”. The basic information of the study population is shown in Table 1. Analysis results showed that there were no differences in these variables including gender, BMI, hypertension (HBP), cerebral infarction, smoking habit and smoking amount. The differences in age and illness years were statistically significant (*p* = 0.0002 and *p* = 0.0063).

**Table 1:** Basic information of the study population

Variables	T2DM patients (*n* = 280)	CIMT ≥ 0.9 (*n* = 66)	CIMT < 0.9 (*n* = 214)	*p*
Gender, (male/female)^a^	181/99	48/18	133/81	0.1160
Age, years^b^	61 (51–71)	66 (56–76)	59 (50–68)	0.0002
BMI, kg/m^2c^	24.64 ± 3.75	25.09 ± 3.52	24.51 ± 3.81	0.2869
Illness years^b^	6 (1–13)	10 (4–25)	6 (0.6–11)	0.0063
Hypertension, *n* (%)^a^	170 (60.71)	46 (69.70)	124 (57.94)	0.0870
Cerebral infarction, *n* (%)^a^	34 (12.14)	11 (16.67)	23 (10.75)	0.1980
Smoker, *n* (%)^a^	78 (27.86)	22 (33.33)	56 (26.17)	0.2560
Daily smoking amount^b^	0 (0–4)	0 (0–12.5)	0 (0–2)	0.3410

*BMI* body mass index

^a^Chi squared test

^b^One-way ANOVA

^c^Kruskal–Wallis equality-of-populations rank test

### Risk analysis of the influence factors of CIMT for T2DM patients

Logistic regression analysis was used to identify the risk factors for CIMT thickening.

All the influence factors such as age, gender, smoking habits and clinical parameters were included in the multivariable model. After adjusting the confounding factors, the results indicated that smoking, UA levels and SDBG were the influence factors for CIMT thickening and the results are shown in Table 2. As illustrated in Table 2, SDBG concentration was closely related to the risk of abnormal CIMT (OR: 281.99, 95% CI 1.24–64,149.02, *p* = 0.042). Smoking was a risk factor for abnormal CIMT. The OR value was 133,860.6 and the differences were statistically significant (*p* = 0.032). The level of 2 h C-peptide was a risk factor for abnormal CIMT and it was statistically significant (OR: 2.65, 95% CI 1.00–7.00, *p* = 0.049). In addition, UA level was an influence factor for CAD (OR: 0.95, 95% CI 0.91–0.99, *p* = 0.047).

**Table 2:** The risk factors leading to CIMT abnormality in T2DM patients

Variables	OR (95% CI)	*p*
Gender	39.95 (0.14–11,145.7)	0.199
Age, years	0.90 (0.74–1.09)	0.274
BMI, kg/m^2^	2.69 (0.99–7.32)	0.052
Illness years	1.17 (0.88–1.54)	0.278
Hypertension	22.70 (0.35–1468.49)	0.142
Cerebral infarction	0.32 (4.86e−15–2.13e+13)	0.944
Smoker	133,860.6 (2.83–6.33e+09)	0.032
Daily smoking amount	0.75 (0.55–1.01)	0.055
HDL, mmol/L	0.01 (2.83e−07–467.55)	0.410
LDL, mmol/L	0.02 (0.0002–1.34)	0.068
Total cholesterol, mmol/L	22.06 (0.33–1497.07)	0.151
Triglycerides, mmol/L	0.75 (0.14–4.20)	0.747
Creatinine, μmol/L	1.00 (0.99–1.00)	0.913
Serum urea, mmol/L	2.71 (0.74–9.97)	0.134
UA, μmol/L	0.95 (0.91–0.99)	0.047
C-peptide level	0.06 (0.0005–5.58)	0.220
0.5 h C-peptide level	0.28 (0.01–6.45)	0.429
2 h C-peptide level	2.65 (1.00–7.00)	0.049
Hba1c	0.53 (0.17–1.63)	0.267
FPG	1.42 (0.73–2.77)	0.305
PPG	1.14 (0.77–1.67)	0.518
HCY, mmol/L	1.33 (0.80–2.21)	0.279
MA/CR	1.00 (0.99–1.00)	0.093
PPGE	0.59 (0.31–1.12)	0.105
LAGE	0.24 (0.05–1.20)	0.081
SDBG	281.99 (1.24–64,149.02)	0.042

*UA* uric acid, *BMI* body mass index, *HDL* high-density lipoprotein, *LDL* low-density lipoprotein, *FPG* fasting plasma glucose, *PPG* postprandial plasma glucose, *HCY* homocysteine, *MA/CR* microalbumin/creatinine ratio, *PPGE* postprandial glycemic excursions, *LAGE* largest amplitude of glycemic excursions, *SDBG* standard deviation of blood glucose, *MAGE* mean amplitude of glycemic excursions

### Blood glucose fluctuation and increased risk of complications

**Fig. 1:**
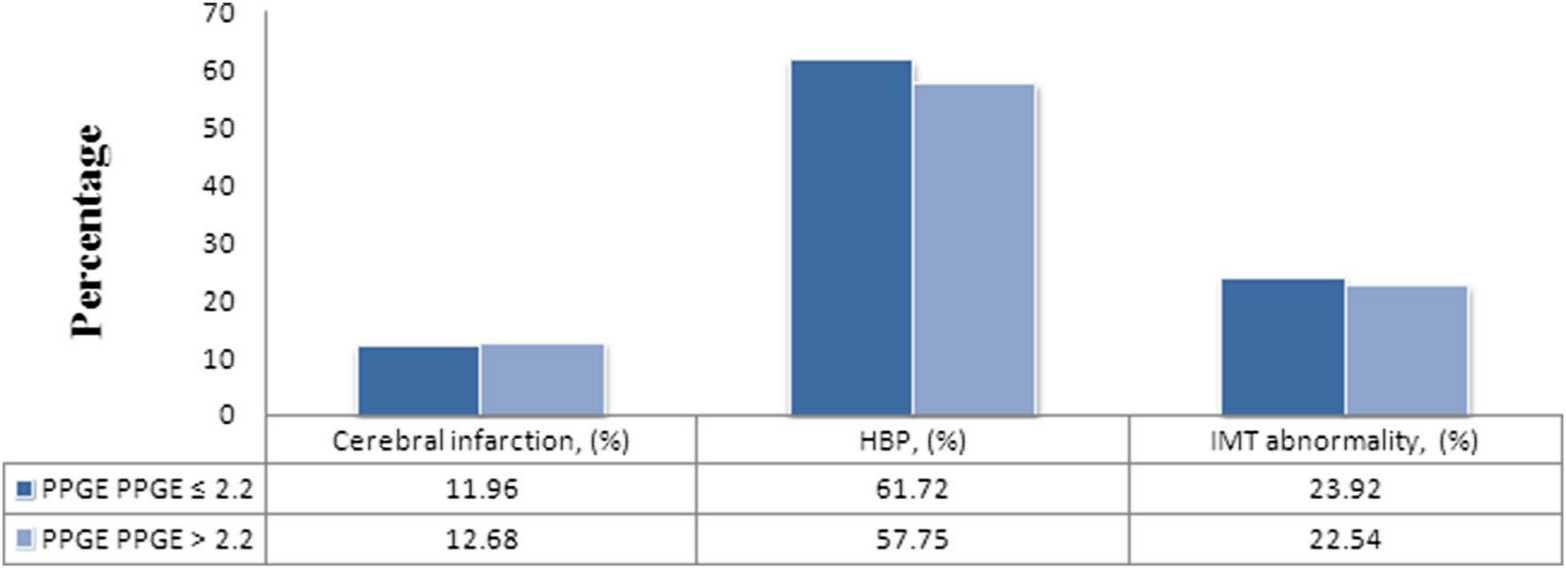
The proportion of complications in different PPGE groups

**Fig. 2:**
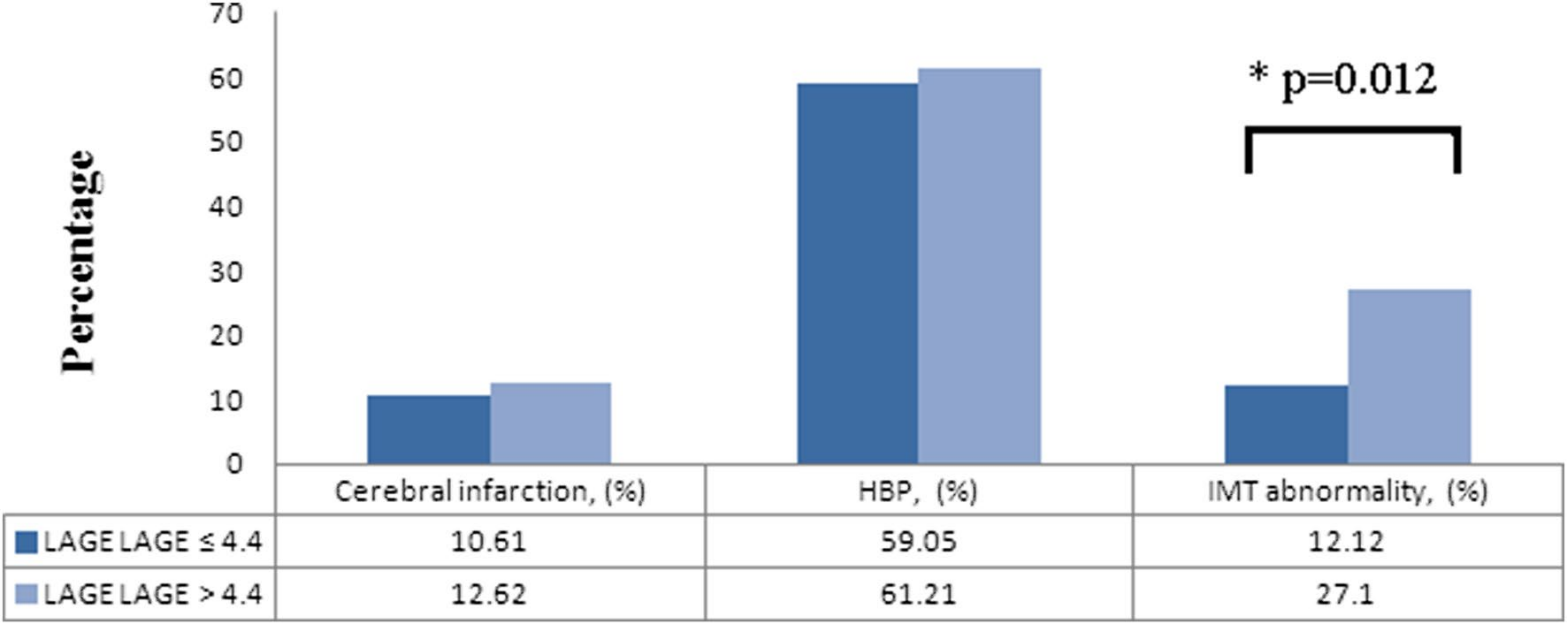
The proportion of complications in different LAGE groups

**Fig. 3:**
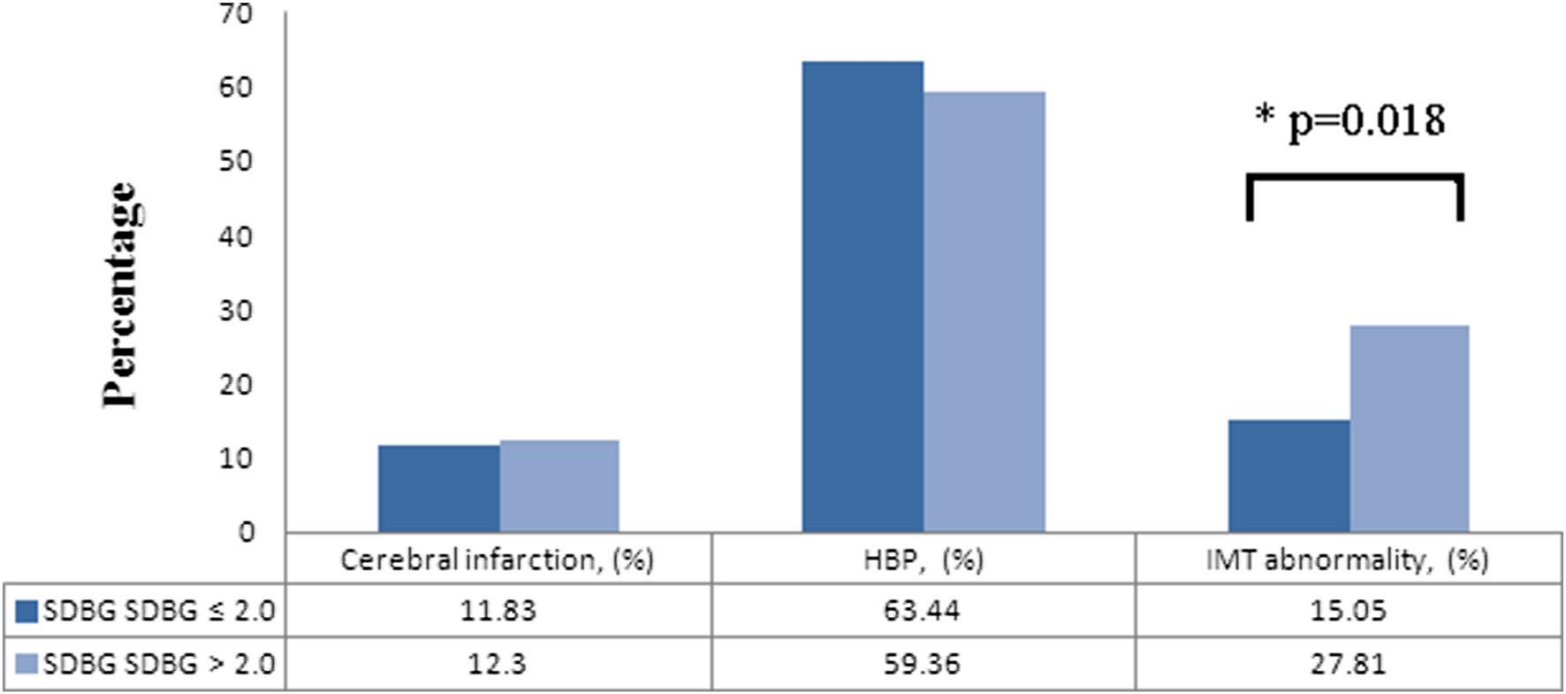
The proportion of complications in different SDBG groups

The “diabetes blood sugar fluctuation management expert consensus” was published by Chinese Medical Association Endocrinology Branch in 2017. PPGE > 2.2, LAGE > 4.4 and SDBG > 2.0 were considered as abnormal blood glucose fluctuations in this consensus. The study population was divided into abnormal blood glucose fluctuation and normal group. Then we compared the differences in major complications of diabetes between the two groups. In this research, no significant differences in the prevalence rates of HBP and cerebral infarction were observed in any blood sugar fluctuation group (Figs. 1, 2, 3). However, a difference in the CIMT abnormal rate could be found in these groups, which were divided by the indexes LAGE and SDBG. In LAGE > 4.4 group, the CIMT abnormal rate was 27.10%, which is higher than 12.12% in the LAGE ≤ 4.4 group. Differences between the two groups were statistically significant (*p* = 0.012, Fig. 2). The CIMT abnormal rate of SDBG > 2.0 group was 27.81%, which is significantly higher than that of SDBG ≤ 2.0 group (*p* = 0.018, Fig. 3).

## Discussion

Previous studies had focused on the harmful effect of high blood sugar and its relationship with complications in diabetes. Traditional risk factors such as smoking also have a significant impact on the abnormal changes in CIMT [24, 25]. In this paper, we investigate the influence of blood glucose fluctuation, clinical parameters and conventional risk factors on CIMT in Chinese Han T2DM patients. The results of this study show that smoking, UA levels, level of 2 h C-peptide and the blood glucose fluctuation parameter SDBG are significantly and independently associated with abnormal CIMT in T2DM patients (*p* = 0.032, *p* = 0.047, *p* = 0.049 and *p* = 0.042, respectively).

According to researches, there exists a close relationship between UA and endothelial dysfunction, which causes the abnormal CIMT and increased risk of CAD [26]. High level of UA was considered as a risk factor for gout and diseases of the cardiovascular system [27]. The main findings of our study suggested that UA level was an influence factor for abnormal CIMT (*p* = 0.047). But the OR value of UA was 0.95, meaning that UA level was a protective factor, which may be caused by a lack of sample size. C-peptide could increase the risk of CAD via a pathway that increases TG or decreases HDL-C levels, which was confirmed by some researches [28, 29]. Our research results were consistent with this.

Blood glucose fluctuation beyond a certain range (PPGE > 2.2, LAGE > 4.4 and SDBG > 2.0) was a risk factor associated with abnormal CIMT. To reveal the relationship between blood glucose fluctuation and diabetic complications, grouping and stratification analysis was conducted. The results indicated that there was no relationship between blood glucose fluctuation and cerebral infarction and no evidence showed that blood glucose fluctuation could directly affect the incidence of HBP. People whose LAGE > 4.4 or SDBG > 2.0 were at high risk of abnormal CIMT. The differences between blood glucose abnormal fluctuation groups and normal groups were statistically significant (*p* = 0.012 and *p* = 0.018).

One limitation of this study is that the sample size is not large enough. The impact of blood glucose fluctuations to abnormal CIMT and incidence of diabetic complications needs larger sample size to be confirmed. In future work, sample size needs to be expanded and more accurate relationships could be explored.

Blood glucose fluctuation beyond a certain range is an independent risk factor associated with CIMT in patients with type 2 diabetes mellitus. T2DM patients, whose blood glucose fluctuation range is wide, should be given more attention to the change of CIMT and cardiovascular system complications. CIMT is a predictor of CAD, which is the most serious complication of diabetes. Early diagnosis and treatment of CAD are beneficial to relieve their pain and improve their quality of life.

## Abbreviations

UA: uric acid
BMI: body mass index
HDL: high-density lipoprotein
LDL: low-density lipoprotein
FPG: fasting plasma glucose
PPG: postprandial plasma glucose
HCY: homocysteine
MA/CR: microalbumin/ creatinine ratio
PPGE: postprandial glycemic excursions
LAGE: largest amplitude of glycemic excursions
SDBG: standard deviation of blood glucose
MAGE: mean amplitude of glycemic excursions
